# Is Hymenotomy Enough for Treatment of Imperforated Hymen?

**DOI:** 10.5812/numonthly.11768

**Published:** 2013-11-13

**Authors:** Alireza Ghadian, Fatemeh Heidari

**Affiliations:** 1 Nephrology and Urology Research Center, Baqiyatallah University of Medical Sciences, Tehran, IR Iran

**Keywords:** Hymen, Imperforate, Hymenectomy

***Dear Editor,***

We read the case report by Rabani and enjoyed it (1). As he said the imperforated hymen is a rare cause of urinary retention in female patients, especially before menarche, but we would like to add some additional comments and suggestions on this issue.

The hymen, which is a mesodermal sheet, should be perforated in the late time of the embryonic period but sometimes it does not happen. This phenomenon may be associated with other anomalies such as genitourinary tract anomalies (renal agenesis) (2) or genetic disorder (ectrodactyly ectodermal dysplasia-clefting syndrome) (3). Imperforated hymen may cause very serious complications, such as acute renal failure or recurrent urinary tract infections (UTIs) and so external genitalia of all female newborns should be examined carefully at birth time. Although usually children remain asymptomatic until menarche, but in rare cases mucus of the vaginal wall do not absorb and will result in hydrocolpos that can cause urinary retention as Rabani reported (1). After menarche, clinical symptoms will appear such as abdominal pain, chronic constipation, low back pain, acute urinary retention (AUR), UTIs and primary amenorrhea (2).

The incidence of AUR due to imperforated hymen is reported between 3 to 46% (4) and is due to compression of the urethra by menstrual products that result in urethral angulation and obstruction. AUR will be treated temporarily by urethral catheterization in all patients. During catheterization, in posterior of introitus a massive bulge can easily be investigated that will prove the diagnosis. If any doubt exists about diagnosis, trans-rectal ultrasonography is very effective in showing hematocolpos. In the cases of complicated obstructive abnormalities, magnetic resonance imaging is very helpful in determining anatomy of urinary tracts (5).

Surgical hymenectomy is the standard treatment of this anomaly and can be done with X, T, cross or crucial incision and resection of excess tissues but sometimes even a vertical incision of hymen may resolve the problem (1, 6). Fortunately almost all patients with urinary retention will cure permanently with standard surgery but in 2011 Sara Abu-Ghanem and her co-workers reported a case of recurrent UTIs after hymenectomy failure in a 14 years old girl (5) and so follow-up is mandatory.

It seems that hymenotomy (a vertical incision in hymen) is enough for resolving the problems but more experience in bigger group is necessary for this result.
